# Epigenetic modifications in muscle regeneration and progression of Duchenne muscular dystrophy

**DOI:** 10.1186/s13148-021-01001-z

**Published:** 2021-01-19

**Authors:** Anna Rugowska, Alicja Starosta, Patryk Konieczny

**Affiliations:** Institute of Human Biology and Evolution, Faculty of Biology, Adam Mickiewicz University, ul. Uniwersytetu Poznańskiego 6, 61-614 Poznan, Poland

**Keywords:** Duchenne muscular dystrophy (DMD), Muscle regeneration, Satellite cells, Epigenetics, Signaling, Pathways

## Abstract

Duchenne muscular dystrophy (DMD) is a multisystemic disorder that affects 1:5000 boys. The severity of the phenotype varies dependent on the mutation site in the *DMD* gene and the resultant dystrophin expression profile. In skeletal muscle, dystrophin loss is associated with the disintegration of myofibers and their ineffective regeneration due to defective expansion and differentiation of the muscle stem cell pool. Some of these phenotypic alterations stem from the dystrophin absence-mediated serine–threonine protein kinase 2 (MARK2) misplacement/downregulation in activated muscle stem (satellite) cells and neuronal nitric oxide synthase loss in cells committed to myogenesis. Here, we trace changes in DNA methylation, histone modifications, and expression of regulatory noncoding RNAs during muscle regeneration, from the stage of satellite cells to myofibers. Furthermore, we describe the abrogation of these epigenetic regulatory processes due to changes in signal transduction in DMD and point to therapeutic treatments increasing the regenerative potential of diseased muscles based on this acquired knowledge.

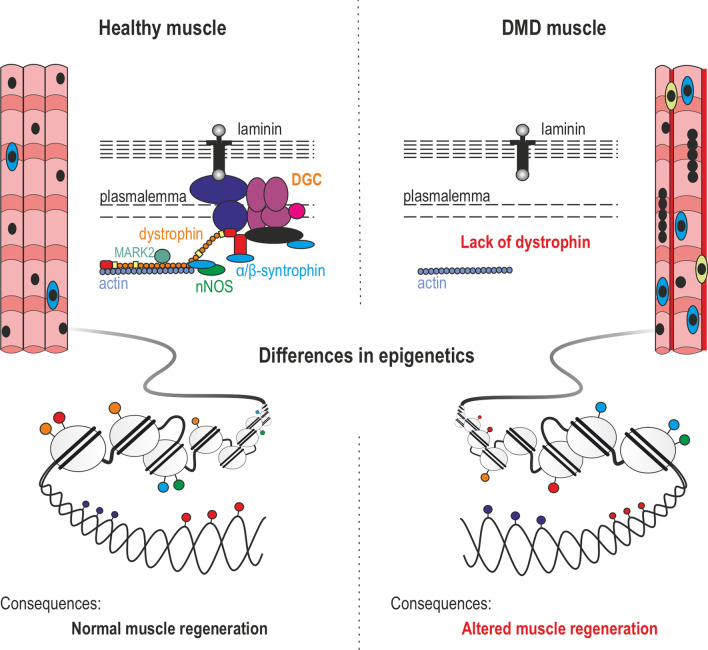

## Introduction

Duchenne muscular dystrophy (DMD, OMIM 310200), the most severe and the most common adult form of muscular dystrophy in humans, is caused by a lack of functional dystrophin due to mutations in the dystrophin gene (*DMD*) [1, 2]. This largest gene in the human genome (> 2.5 Mbp) gives rise to several transcripts that encode protein isoforms ranging in size from 40 to 427 kDa, which are variously distributed in many cell types [3–11]. Because *DMD* is located on the X chromosome (Xp21.2 region), the disease primarily affects boys with estimates of incidence ranging from one in every 3500 to more recent estimates of 1:5000 live births [12–14]. Interestingly, females also suffer from DMD in rare instances (1:50,000,000 live births) [15] and approximately 8% of female mutation carriers experience muscle weakness [16]. The genetic defects in the *DMD* gene include deletions (65%), duplications (5–10%), and point mutations (10–15%) [17–19].

**Fig. 1:**
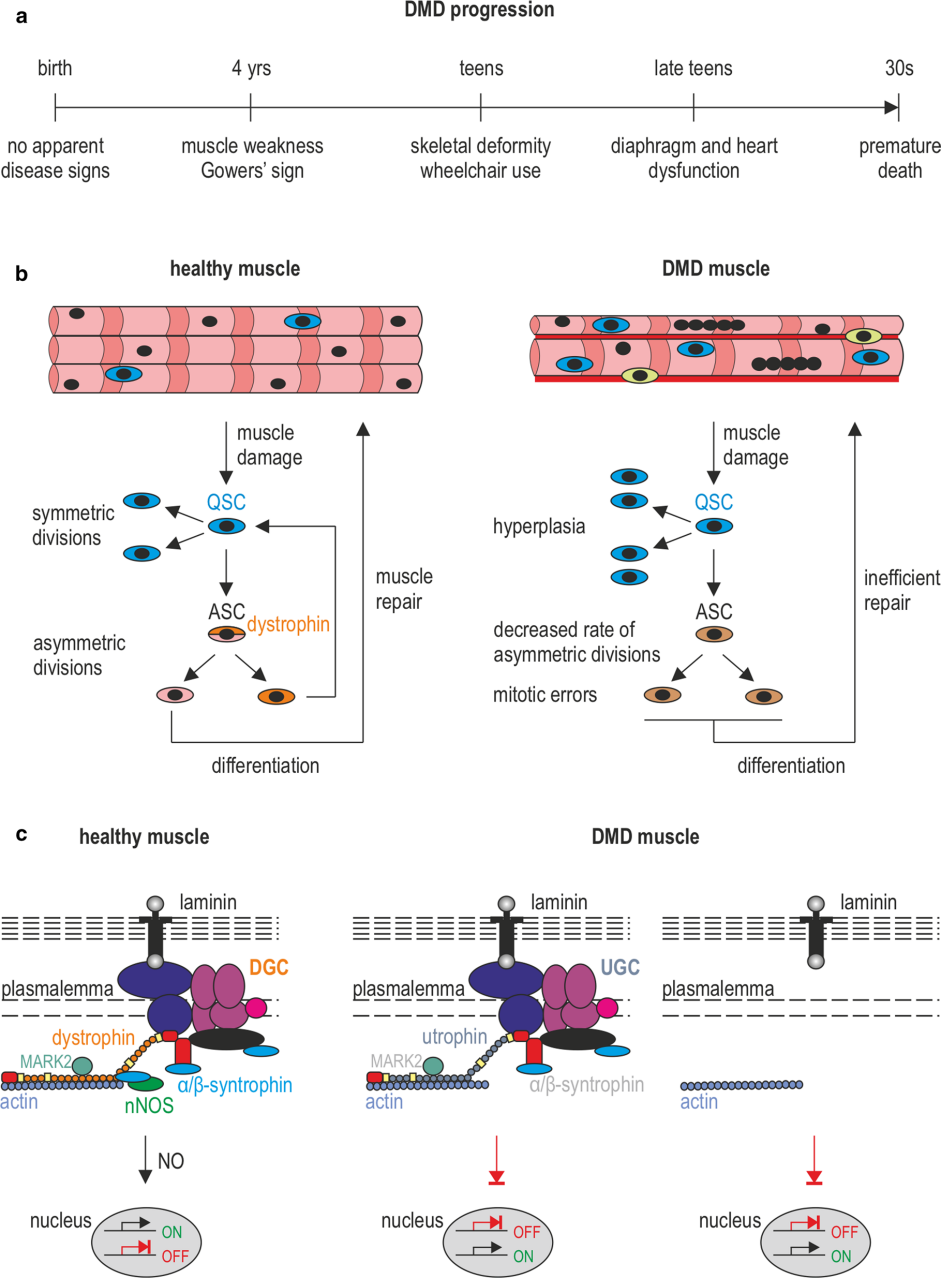
DMD—the disease of satellite cells and myofibers. **a** A timeline showing the progression of DMD symptoms. The affected boys develop motor skills until the age of 4–6, however, at a lower rate than their peers. Muscle weakness and Gowers’ sign are apparent from the age of 4. The condition of the muscles deteriorates quickly and the patients are forced to use a wheelchair in their teens. Typically, in the late teens, they need to start to use a temporary and then 24-h ventilation aid as a consequence of dysfunctional respiratory muscles. The boys die usually in their twenties/thirties, due to respiratory or cardiac failure. **b** In response to damage, QSCs (marked sky blue) that reside between the basal lamina and the plasmalemma, are activated and divide asymmetrically to generate SCs that return to the quiescent state (marked orange) and SCs undergoing differentiation into myoblasts that participate in muscle repair (marked pink). The asymmetric division is driven by dystrophin in combination with its binding partner, MARK2 (see in **c**). Lack of dystrophin leads to diminished levels of MARK2 and β-syntrophin in satellite cells (and α-syntrophin in skeletal muscle and NMJs), lower amounts of asymmetric divisions, and an increase in abnormal mitotic divisions. Also, note the elevated numbers of satellite cells in DMD muscles that are generated through symmetric divisions as well as increased fibrosis (marked red) and infiltration of immune cells (marked yellow). **c** DGC in the plasmalemma of satellite cells and myofibers performs structural and signal transduction functions, including those that pertain to NO production. In DMD muscles, the loss of dystrophin results in partial compensatory assembly of the utrophin-based complex (UGC) as well as other proteins and protein complexes (not shown). In neither case, the correct signal transduction functions are restored

The first sign of the disease is muscle weakness, which starts in boys at around the age of four and progresses quickly (Fig. 1a). It is usually accompanied by a loss of muscle contraction in the thigh and pelvis muscles followed by arm muscle weakness. At this point, the classic DMD symptoms begin to emerge, i.e., Gowers’ sign (patients use their hands and arms to ‘walk-up’ their body to stand from a sitting or squatting position), rocking gait, or walking on toes. In the second phase of the disease progression, movement causes increasing problems as patients experience difficulty walking, climbing stairs, or getting up from the floor. At this stage, cooperation with physiotherapists is needed, but also with a psychologist who will prepare the child for loss of mobility. As a result of the disease progression, the function of the lower extremities deteriorates and it becomes more difficult for the patient to maintain the correct body position. With time, the affected boys are forced to use a wheelchair. The consequence of the fragility and progressive loss of DMD myofibers is the accumulation of fibrotic and adipose tissue, thus contributing to skeletal muscle mass loss and function [20]. Ultimately, DMD leads to premature death in the twenties or thirties due to diaphragm dysfunction or cardiac failure [21, 22].

The skeletal muscle has the intrinsic ability to regenerate damaged myofibers after injury or as a consequence of a disease process. High regenerative capacity is directly linked to the presence of satellite cells (SCs) (Fig. 1b) [23], which are undifferentiated skeletal muscle precursor cells residing in a niche between the muscle fiber membrane (sarcolemma) and the basement membrane surrounding each muscle fiber [24–27]. In principle, these cells are required not only for myofiber regeneration but also for the growth and maintenance of skeletal muscle. SCs are normally mitotically quiescent (QSCs) but are poised to act and enter the cell cycle in response to stress stimuli such as injury [28]. Activated satellite cells (ASCs) undergo asymmetric division, myogenic differentiation, and self-renewal to restore the pool of QSCs (Fig. 1b, left panel). Defects in SCs have been shown to contribute to the etiology of some muscle diseases [29]. Specifically, DMD progression has been linked to a failure of SCs to divide asymmetrically and maintain the damage-repair cycle (Fig. 1b, right panel). This is due to the fact that in the dystrophin–glycoprotein complex (DGC), dystrophin is associated with the serine–threonine protein kinase 2 (MARK2), which plays a pivotal role in establishing cell polarity (Fig. 1c). Moreover, over time, the SC pool in diseased tissues undergoes exhaustion [30, 31] and cannot replenish damaged myofibers as underscored by the progressive loss of muscle mass in DMD patients. Inflammatory processes downstream of dystrophin deficiency, as well as metabolic abnormalities and defective autophagy, additionally contribute to muscle pathology in DMD. Particularly, chronic inflammation caused by muscle damage in DMD patients has an important impact on disease progression [32, 33].

The structural role of dystrophin is closely related to its participation in the DGC, which can be subdivided into three smaller subcomplexes: the dystroglycan complex (α- and β-dystroglycan, generated by proteolytic cleavage of a single precursor protein); the sarcoglycan complex (α-, β-, δ-, γ-sarcoglycan); and the complex located in the cytoplasm (Fig. 1c) [34]. The generally accepted role for the DGC is to stabilize the plasma membrane during muscle contraction, signified by the fact that disruption of the linkage between the cytoskeleton and the extracellular matrix (ECM) occurs in DMD [35–37]. Furthermore, the DGC also plays a pivotal role in the organization of neuromuscular junctions, where it stabilizes the postsynaptic machinery, including receptors for the neurotransmitter acetylcholine [38, 39]. However, the role of dystrophin is not limited to the structural function. Growing evidence suggests that dystrophin, through its multiple protein connections, plays a major role in gene expression via regulating signal transduction (Fig. 1c), including pathways that activate nitric oxide (NO) production, Ca^2+^ entry, and the production of reactive oxygen species (ROS) [40].

Despite the undoubted progress in the development of experimental therapeutic approaches, DMD is still incurable. Symptomatic treatment of the progressive loss of muscle tissue, caused by myofiber degeneration and their inefficient regeneration, is currently limited to corticosteroids that alleviate secondary inflammatory processes in DMD. In recent years, it has become more evident that epigenetic mechanisms such as DNA methylation or histone modification have a pivotal role in regulating muscle regeneration and regenerative medicine is providing novel therapeutic strategies by developing epigenetic drugs aimed to manipulate the chromatin targets of individual signaling pathways. In this review, we discuss the epigenetic regulation of various stages of skeletal muscle regeneration and the interplay of numerous factors that define the specific state of epigenetic homeostasis in health and DMD.

## Epigenetics: the basics

The term *epigenetics* was first used by a British embryologist, Conrad H. Waddington, in 1942 [41]. At that time, *epigenesis* referred to the differentiation of cells from their initial totipotent state during embryonic development. Today, the term *epigenetics* indicates a field of science that studies the relationship between the genetic code and the living environment—mental and physical and, more specifically, that describes the mechanisms and effects of biochemical modification of genome expression without changing the DNA sequence [42]. Genome expression can be modified by environmental factors, lifestyle, upbringing, or emotions, and to some extent, these modifications can be inherited. At any stage of human life, from conception to death, intracellular stimuli and environmental factors such as nutrition, physical activity, environment pollution, stress, and bacterial infections also regulate the expression of our genes [43, 44].

**Fig. 2:**
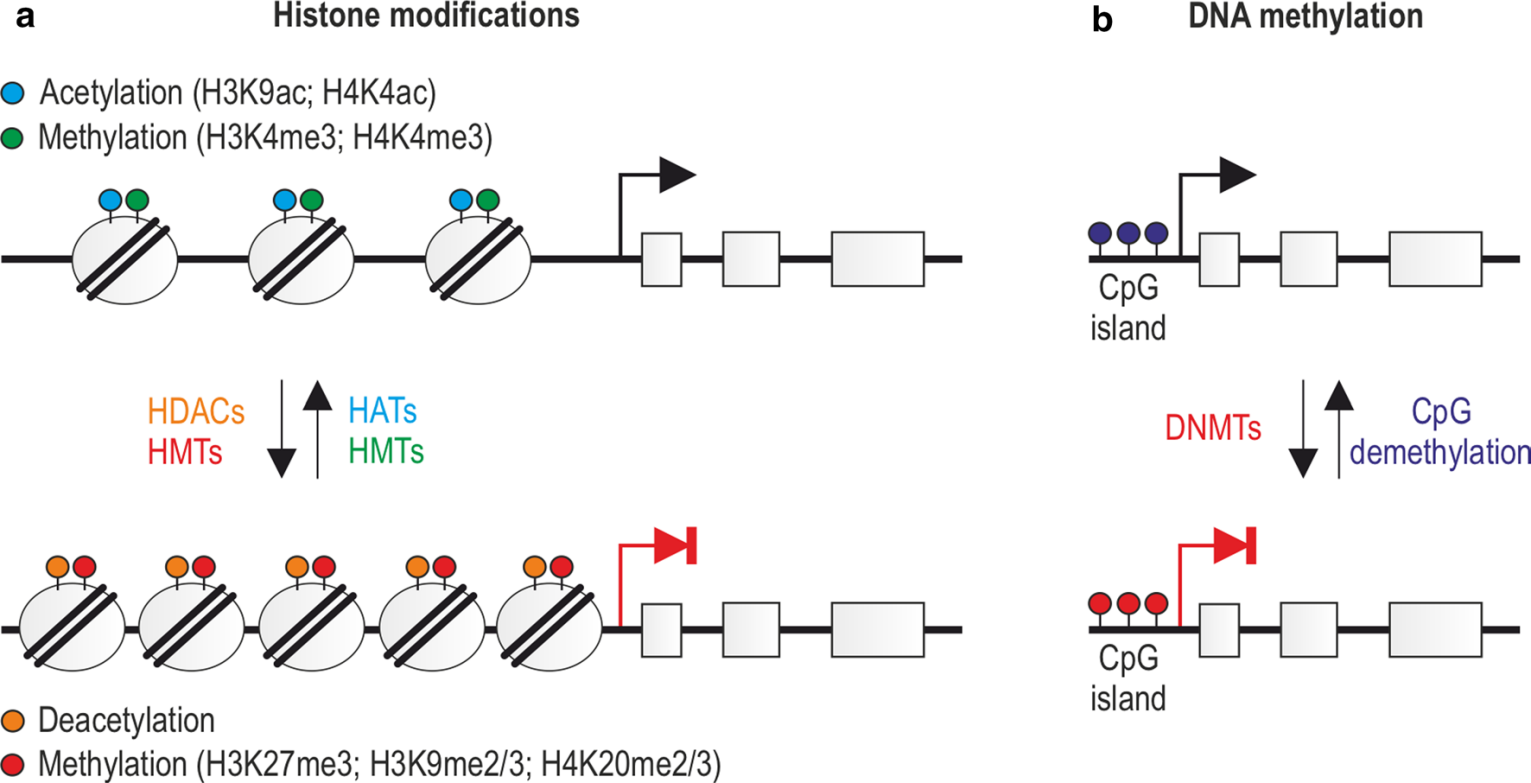
Basic principles of epigenetic modifications. **a** Epigenetic mechanisms regulating gene expression via histone modifications. Chromatin transcription permissive histone modifications such as H3K9ac/H4K4ac (acetylation) and H3K4me3/H4K4me3 (methylation) are catalyzed by HATs and HMTs, while repressive, including H3K27me3, H3K9me2/3, H4K20me2/3 (methylation), and deacetylation, through the action of HMTs and HDACs. Histone modifications and enzymes catalyzing the corresponding reactions are marked with matching colors. **b** Epigenetic mechanisms regulating gene expression via DNA modifications. Methylation and demethylation of CpG islands in the promoter regions of genes cause gene silencing and transcriptional activation, respectively. DNMTs and the process of CpG demethylation are, respectively, color-matched to methylated and demethylated CpGs

**Fig. 3:**
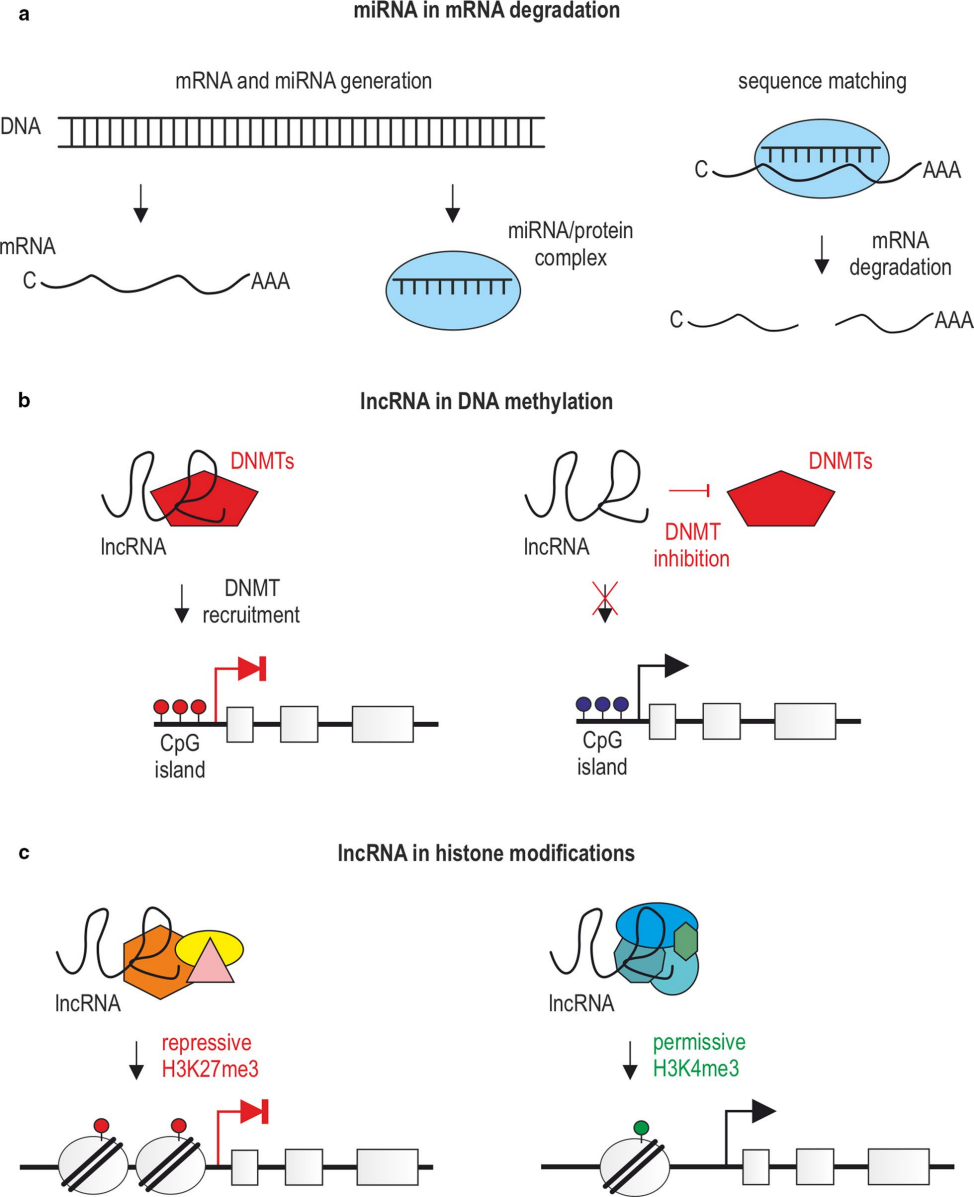
Noncoding RNAs in chromatin modifications. **a** miRNAs induce degradation of target mRNAs containing matching sequences via the RNA–protein complex. **b**, **c** lncRNAs can recruit or inhibit various enzymes or protein complexes to induce transcription repressive and permissive DNA (**b**) or histone modifications (**c**)

In general, the major epigenetic signals include modifications related to (1) covalent posttranslational reversible modifications of histone proteins, such as methylation, acetylation, phosphorylation, ubiquitination, or incorporation of histone variants (Fig. 2a), (2) DNA methylation and demethylation (Fig. 2b), and (3) gene regulation by noncoding RNAs (ncRNAs), (Fig. 3) [45]. It is important to realize that epigenetic changes occur naturally in normal development and health as well as in aging and disease. Below we describe the selected epigenetic mechanisms.

### Histone modifications

Chromatin activity is regulated by chromatin-modifying multiprotein complexes, whose catalytic subunits induce reversible posttranslational histone modifications such as acetylation, methylation, phosphorylation, or ubiquitination and are associated with permissive and repressive chromatin states [46–51]. Among them, histone acetylation and methylation are the most common mechanisms in myogenesis regulation, while phosphorylation and ubiquitination occur less frequently. In general, acetylation of lysine residues of histones H3 and H4 (H3K9ac, H4K4ac) and trimethylation of lysine 4 of histones H3 or H4 (H3K4me3, H4K4me3) are associated with activation of transcription (permissive chromatin), whereas trimethylation of lysine 27 of histone H3 (H3K27me3), di-/trimethylation of lysine 9 of histone H3 (H3K9me2/3), and di-/trimethylation of lysine 20 of histone H4 (H4K20me2/3) cause gene repression by chromatin condensation (Fig. 2a) [52].

Histone acetylation is dynamically regulated by the antagonistic action of histone acetyltransferases (HATs) and histone deacetylases (HDACs) (Fig. 2a) that operate as catalytic centers of multiprotein assemblies. HATs are classified based on their cellular localization in the nucleus (type A) and the cytoplasm (type B). While not much is known about cytoplasmic HATs, several nuclear HATs have been identified and further divided into three major families that differ in their primary structure homology, represented by (1) p300/CBP-associated factor (PCAF), (2) the p300/CBP family, including p300 and cAMP response element-binding (CREB) protein (CBP), and (3) the MYST family [53, 54]. Generally, histone acetylation is achieved by the addition of an acetyl group (–CH3CO) transferred from acetylcoenzyme to one or more lysine residues in the ϵ-amino group of histones, resulting in the relaxation of chromosomal DNA [55]. Conversely, mammalian HDAC enzymes remove the acetyl group from histone proteins, causing chromosomal DNA to be less accessible to transcription factors [56, 57]. There are currently 18 known human HDACs grouped into four classes. Classes I, II, and IV are zinc-dependent HDACs, while class III (also called Sirtuins, SIRT) comprises HDACs that require NAD^+^ [58]. Interestingly, class I and IV HDACs are predominantly sublocalized in the nucleus, whereas class II HDACs shuttle between the nucleus and the cytoplasm [59]. Similar to class II, class III HDACs can occur in the nucleus and cytoplasm; however, enzymes from this class can also act in the mitochondria [60].

Histone methylation occurs on lysine and arginine residues and is generated by the activity of histone lysine methyltransferases (HKMTs), (Fig. 2a), and protein arginine methyltransferases (PRMTs). The first group includes, among others, the following families: suppressor of variegation 3–9 (SUV39), e.g., G9a methyltransferase that methylates lysine 9 of histone H3 (H3K9); enhancer of zeste homolog (EZH); SET1, which includes lysine methyltransferase 2A (MLL); SET2; SET7 and suppressor of variegation 4–20 (SUV4-20). The group of arginine methyltransferases comprises of ten mammalian PRMTs (PRMT1-10) that have been identified to date [61]. Here, PRMT4 or coactivator-associated arginine methyltransferase 1 (CARM1) was the first PRMT characterized as an activator of transcription by methylating histone H3 [62]. Conversely, the methyl groups from histones are removed by the action of two classes of histone demethylases (HDMs; not shown) [63], a lysine-specific histone demethylase 1 (LSD1), which has mono- and di-demethylating histone H3 activity (H3K4, H3K9) and the Jumonji C (JmjC) domain-containing family of HDMs, which unlike LSD1, is capable of removal of trimethylation [64].

**Fig. 4:**
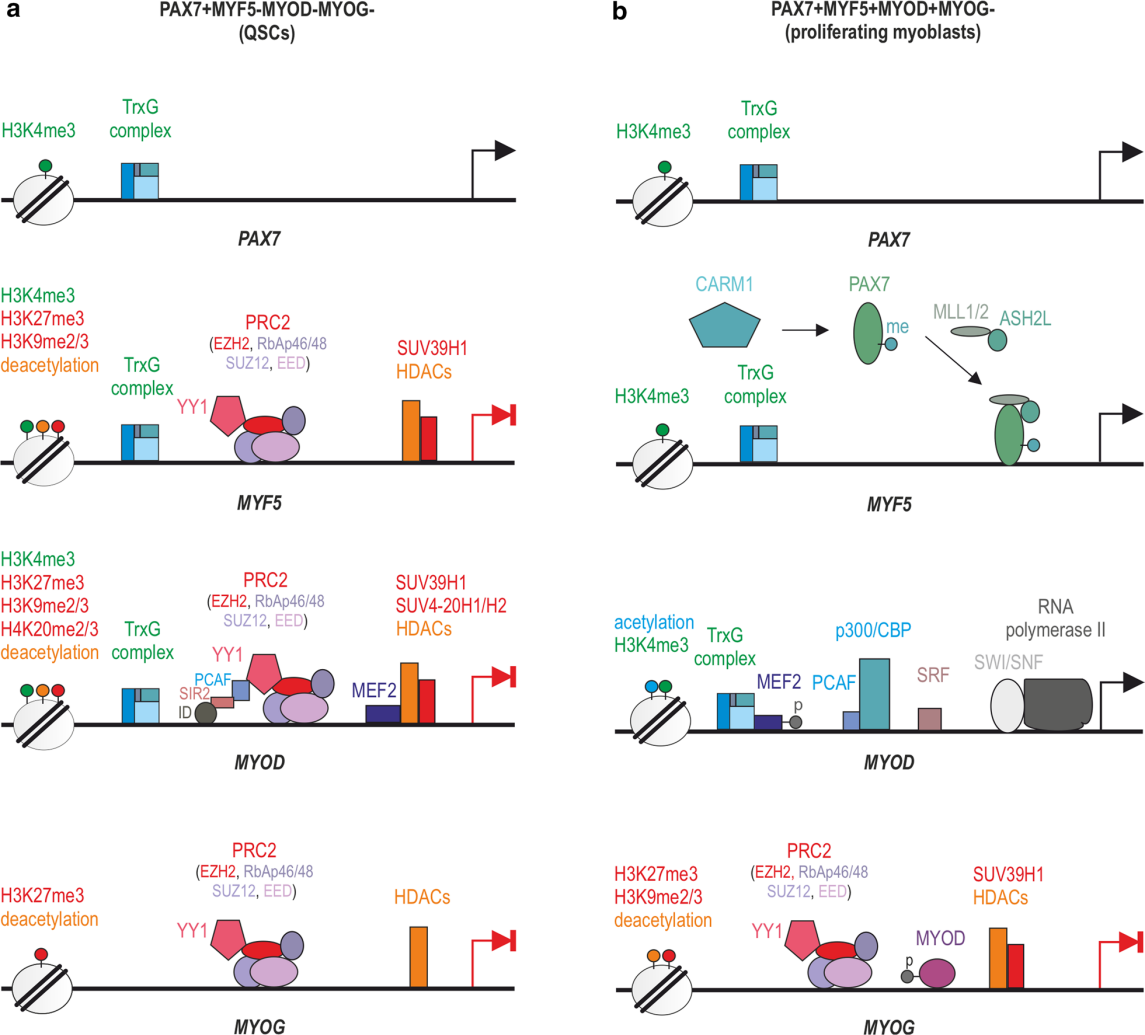
Epigenetic regulation of the quiescent and proliferative state of SCs. **a** In QSC and during self-renewal, the *PAX7* promoter is active, holding permissive chromatin marks through the TrxG activity, while *MYF5/MYOD* expression is repressed despite containing both permissive and repressive marks, including H3K4me3 (TrxG) and H3K27me3 (PRC2, YY1-EZH2 complex), respectively. Repression of *MYF5* and *MYOD* is also induced by SUV39H1 (H3K9me2/3) and by removal of acetylation marks carried by HDACs. Furthermore, note that *MYOD* expression is repressed by ID, SIR2 as well as MEF2 and SUV4-20H1/H2 (H4K20me2/3). The *MYOG* gene expression is inactivated via the cooperative action of PRC2 and HDACs. **b** In proliferating myoblasts, CARM1 targets and methylates PAX7 protein, which facilitates recruitment of TrxG to the *MYF5* promoter. In addition, PRC2 and HDACs are removed from the *MYOD* promoter, which enables binding of TrxG, PCAF-p300/CBP, SRF and phosphorylated MEF2 as well as the SWI/SNF complex that induces chromatin relaxation and *MYOD* transcription. Also note that at this stage of myogenesis, phosphorylated MYOD as well as SUV39H1 halt expression of *MYOG* in addition to PRC2 and HDACs

Functional protein complexes are often required for HMTs to exert their catalytic activities, for example polycomb repressive complex 2 (PRC2) targets H3K27me3 addition to developmentally regulated genes. In humans, PRC2 consists of four core subunits required for its optimal functioning: EZH1 or EZH2, suppressor of zeste 12 (SUZ12), embryonic ectoderm development (EED), and retinoblastoma suppressor-associated proteins 46/48 (RbAp46/48) (Fig. 4). EZH1 and EZH2 are Su(var)3–9, enhancer of zeste, and trithorax catalytic (SET) domain-containing proteins harboring HKMT activity, while SUZ12 and EED are involved in the PRC2 stability and are required for the EZH1/2 catalytic activities [65, 66]. In turn, RbAp46/48 that constitute the fourth core subunit of PRC2 are histone chaperones that play a pivotal role in establishing and maintaining the chromatin structure and are not required for the enzymatic activity of EZH [67, 68]. Moreover, protein complexes that catalyze the repressive state of chromatin often cooperate. Boros et al., for example, proposed a model, in which H3K27me3-bound PRC2 stabilizes H3K9me3-anchored heterochromatin protein 1α (HP1α), a structural adapter necessary to form and maintain a condensed structure of heterochromatin in two ways: directly by interaction with SUZ12 or indirectly through an unknown factor [69], as was recently suggested by Canzio et al. [70]. In contrast, the main complex involved in permissive chromatin changes, the trithorax group protein (TrxG), is most commonly linked to gene activation by inducing H3K4me3 due to MLL1/2 HKMT activity (Fig. 4) [71].

**Fig. 5:**
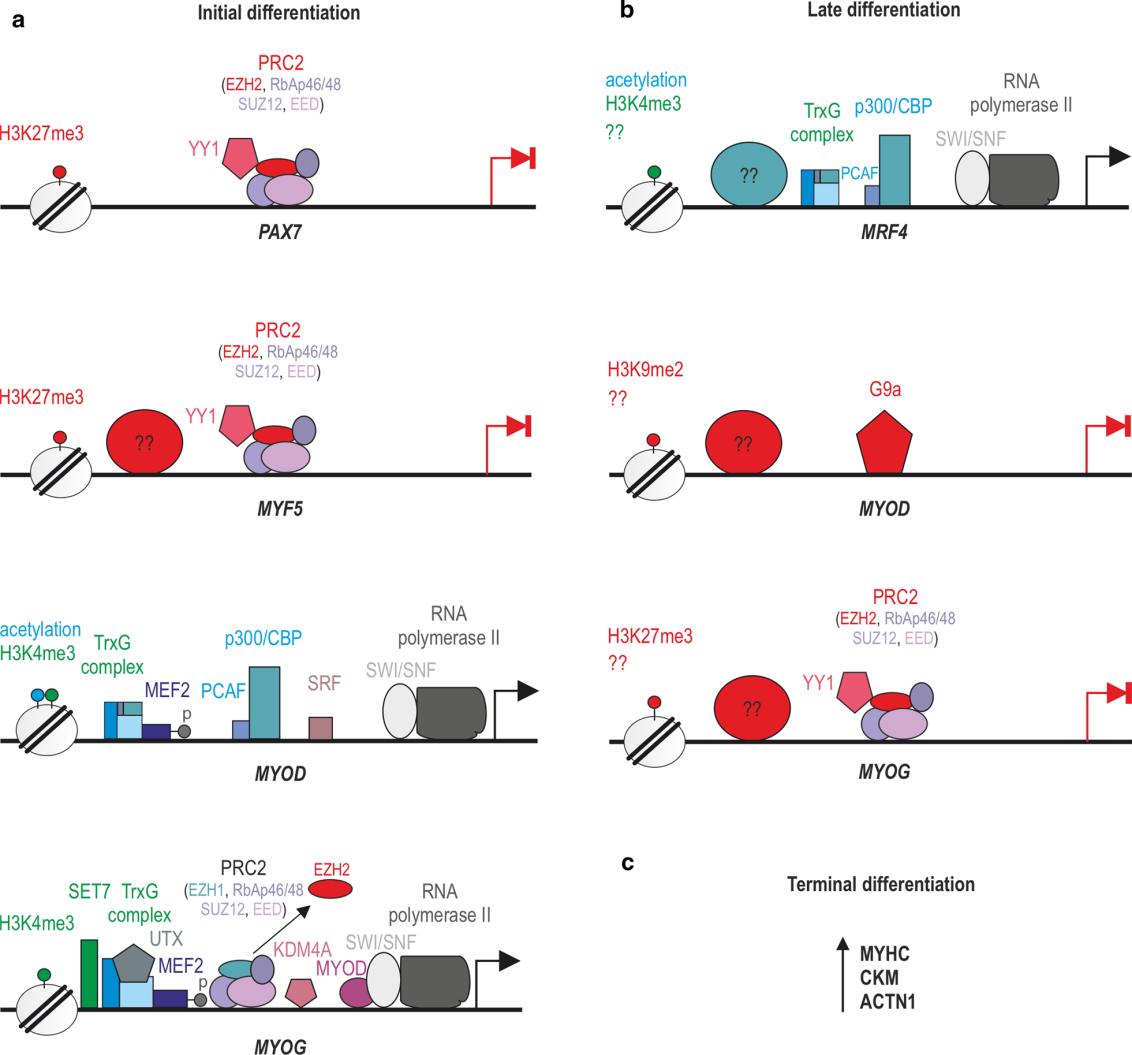
Epigenetic regulation in differentiating myogenic cells. **a** In the initial stage of differentiation, *PAX7* and *MYF5* expression is inhibited via H3K27me3 catalyzed by PRC2 (YY1-EZH2 complex) and additionally, other unknown factors (inhibition of *MYF5*). Conversely, *MYOD* gains active transcription marks (H3K4me3, acetylation) through the combined action of TrxG, MEF2, PCAF-p300/CBP, SRF, as well as the SWI/SNF complex. The high level of MYOD contributes to an increase in the production of MYOG and via the action of KDM4A, TrxG, phosphorylated MEF2, UTX, SET7 and also by SWI/SNF complex. Additionally, EZH1 must be present on the *MYOG* promoter to enable its transcription. The combined action of MYOD and MYOG leads to the expression of genes characteristic for late differentiation, such as *MRF4* **(b)**, then levels of MYOD and MYOG decrease in response to the G9a HKMT and the PRC2-YY1 complex, respectively, and due to other unknown factors. **c** In the terminal differentiation, the level of MRF4 remains high and proteins characteristic for mature skeletal muscle such as MYHC, CKM, and ACNT1 are generated

Changes in chromatin conformation require energy, which is obtained during the ATP hydrolysis reaction. In the remodeling process that activates transcription, the DNA-histone contact is loosened, allowing the nucleosomes to move along a specific DNA sequence [72, 73]. An important multisubunit enzymatic complex involved in this process is the SWItch/sucrose non-fermentable (SWI/SNF) (Figs. 4b and 5), which in humans consists of complexes that contain either Brahma (*BRM*) or Brahma-related gene 1 (*BRG1*) ATPases, associated with BRG1-associated factors (BAFs) [74]. The function of the SWI/SNF complex is to form the RNA polymerase II preinitiation complex that promotes transcriptional elongation. In detail, the ATPase subunits contain bromodomains that can recognize acetylated lysine on histone proteins and are responsible for nucleosome remodeling [75]. SWI/SNF enzymes can also physically interact with HATs and HDACs, showing the potential for coordination of chromatin remodeling activities [76].

### DNA methylation

DNA methylation is a heritable yet reversible epigenetic modification and increasing evidence shows that methylated DNA is an important regulator in many biological processes, including X-chromosome inactivation, genomic imprinting, and gene expression [77–82], signified by the fact that aberrant DNA methylation patterns are often observed in many diseases [81]. In mammalians, the most often methylated nucleotide is cytosine (5-methylcytosine) in cytosine-guanine (CpG) adjacent sites (Fig. 2b) and also adenine at the 6-nitrogen position of the purine ring in the symmetric tetranucleotide motif 5′-G-A-T-C-3′ [82]. In adult mammals, palindromic CpGs of both DNA strands are methylated at the level of 3.5–4.5% in a cell- and tissue-dependent manner [83, 84]. Interestingly, about 70% of promoters within the human genome contain CpG sequences and their methylation is related to gene expression silencing (Fig. 2b). Additionally, asymmetric methylation at non-GpG sites is also observed [85–87].

The methyl mark is deposited by DNA methyltransferases (DNMTs), enzymes that transfer a methyl group from S-adenosyl-L-methionine (SAM) to the cytosine at the 5′ position (Fig. 2b) [88]. To date, three active methyltransferases have been identified in humans: DNMT1, DNMT3A, and DNMT3B. All of them require accessory proteins for their biological function, such as ubiquitin-like containing plant homeodomain (PHD) and ubiquitin-like with PHD and ring finger domain 1 (UHRF1) or DNMT3-like (DNMT3L) [89]. Interestingly, the previously considered methyltransferase, DNMT2, turned out not to methylate DNA, but instead position 38 in aspartic acid tRNA [90]. Thus, to better reflect its biological function, its name has been changed from DNMT2 to TRDMT1 (tRNA aspartic acid methyltransferase 1) [91]. DNMT3L was also previously considered as a DNA methylating enzyme, related to DNMT3A and DNMT3B in structure; however, unlike the other DNMTs, it does not possess any inherent enzymatic activity, despite its critical role in the DNA methylation process [92]. Human DNMT1 has a high preference for hemimethylated DNA and thus is called maintenance DNA methyltransferase, while DNMT3A and DNMT3B can place methylation marks on previously unmethylated CpGs and thus are mainly responsible for the de novo DNA methylation [93, 94]. Importantly, a relation between DNA methylation and the histone-modifying machinery was observed. Specifically, methyl-CpG-binding domain (MBD) proteins, which are necessary to recognize methylated CpGs, recruit HDACs and HMTs (e.g., PRC2) to methylated DNA regions [95, 96].

### Gene regulation by noncoding RNA

The expression of genetic information may be affected by a specific group of regulatory molecules, so-called regulatory noncoding RNAs (rncRNAs) (Fig. 3) [97]. As the name indicates, rncRNAs do not encode proteins; however, often control gene transcription and can also directly influence the structural properties of chromatin via alterations in DNA methylation and histone modification [98]. Importantly, their functional roles are signified by the fact that their expression is strictly controlled and depends on the developmental stage as well as the differentiation level of the cell [99, 100]. The classification of rncRNA molecules is based on their size, and thus, a distinction can be made between small noncoding RNAs (sncRNAs; < 200 nt) and long noncoding RNAs (lncRNAs; > 200 nt). sncRNAs include microRNAs (miRNAs), piwiRNAs (piRNAs), or short interfering (siRNAs) [101, 102]. In particular, miRNAs (18–25 nt long) are highly conserved molecules across species that act as negative regulators of about 60% of mRNAs through their degradation (Fig. 3a) [103–106].

miRNAs can be viewed as a part of a larger genome expression feedback loop as they target the expression of key epigenetic enzymes such as DNMTs, HDACs, or EZHs [107, 108] and, on the contrary, the expression of miRNAs is regulated by the epigenetic machinery, such as DNA methylation, RNA, and histone modifications [109]. In contrast to miRNAs, piRNAs are involved in de novo DNA methylation [110], while siRNAs are necessary for the RNA-induced transcriptional silencing (RITS) ribonucleoprotein complex to be located in a specific region of chromatin, which leads to the formation of heterochromatin by cytosine and H3K9 methylation [111].

The most accepted categories of lncRNAs are sense and antisense, transcribed on the same or opposite strand of a gene; intronic; intragenic arising from an intron of a protein-coding gene or a region located between two protein-coding genes, and circular RNAs (circRNAs) [112]. Based on their location, lncRNAs can be also distinguished as nuclear or cytoplasmic [113]. In the nucleus, lncRNAs play a crucial role as modifiers of chromatin where they are involved in the spatial localization of DNA-associated proteins to genomic loci, positioning of nucleosomes, and formation of chromatin loops [114]. For instance, direct physical associations between lncRNAs and DNMTs have been attributed to gene expression inhibition [115, 116] or transcription enhancement (Fig. 3b) [117]. Furthermore, lncRNAs can also affect the chromatin structure through the interaction with chromatin-modifying complexes catalyzing repressive H3K27me3 or permissive H3K4me3 marks (Fig. 3c).

## Myogenesis and epigenetic regulation of muscle gene expression

Myogenesis is the formation of muscle tissue, either during embryonic development or in response to myofiber damage that is observed in DMD patients (Fig. 1b) [118]. In the latter case, the myogenic process can be distinguished into three different stages: (1) an inflammatory process involving macrophages, (2) activation and division of SCs, and (3) formation and development of new muscle fibers [119]. In the first stage, leukocytes, neutrophils, and then macrophages, which play the most important role in the initial phase of regeneration, start to accumulate at the site of the damage. There are two subpopulations of macrophages, M1 (pro-inflammatory) and M2 (anti-inflammatory) [120]. In the M1 group, the cells show expression of neural cell adhesion molecule 1 (CD65) protein, secrete pro-inflammatory cytokines such as tumor necrosis factor alfa (TNF-α) and interleukin 1 beta (IL-1β), and are responsible for the removal of damaged fiber fragments during phagocytosis. Macrophages of the M2 group show expression of CD163 protein and secrete anti-inflammatory cytokines, interleukin 10 (IL-10), among others, which inhibits further development of the inflammatory process [121]. Additionally, macrophages from this group stimulate the activation, proliferation, and division of SCs, which initiates the next stage of muscle fiber regeneration [122]. Generally, on the second day after damage, QSCs defined by the expression of transcription factor paired box 7 (*PAX7*) become activated, start to multiply, and then divide and differentiate to create new skeletal muscle fibers. Interestingly, SCs proliferation and migration to the regeneration site have been observed along the entire fibers of injured muscle [123].

It is noteworthy that in the regenerating muscle, the largest pool of SCs consists of cells that express *PAX7* and myogenic factor 5 (*MYF5*) (*PAX7*+/*MYF5*+). These cells divide, differentiate into myocytes, and either generate new fibers de novo or fuse and repair the damaged fiber (Fig. 1b). However, there is also a population of SCs that expresses *PAX7* but is *MYF5* negative (*PAX7*+/*MYF5*−). This type of cell undergoes symmetric and, upon activation, asymmetric division (Fig. 1b). In the latter case, the division results in two daughter cells expressing either *PAX7*+/*MYF5*+ (progenitor cells capable of the differentiation process; Fig. 4b) and cells expressing *PAX7* without *MYF5* (*PAX7*+/*MYF5*−; Fig. 4a). *PAX7*+/*MYF5*- cells retain undifferentiated stem cell properties and are responsible for renewing the population of QSCs [124].

### Epigenetic regulation of the quiescent state of SCs

SCs maintain and regenerate the damaged skeletal muscle tissue. Initially, it was thought that sustaining SCs at rest was the result of a lack of nutrients or extracellular signals that could induce cell proliferation. This dogma has changed radically as it was noted that quiescence is an active and reversible state, controlled by specific cellular epigenetic mechanisms [125]. The chromatin permissive and repressive epigenetic processes acting in SCs have been defined in the context of the expression (or lack of it) of specific transcription factors, some of which include myogenic regulatory factors (MRFs). MRFs are not expressed in QSCs but occur in an orderly and coordinated manner during the myogenesis process in ASCs, proliferating myoblasts and in mature muscle cells that fuse to form regenerated skeletal muscle fibers [126].

#### DNA methylation

DNA methylation has classically been postulated as one of the major repressive systems acting on the muscle gene loci. A recently published dataset of the whole transcriptome from QSCs and proliferating SCs showed downregulation of Dnmt3a (de novo DNMT) during activation of SCs, while the expression of another methyltransferase, *Dnmt1* (maintenance DNMT), was increased [127]. These observations suggest that specific DNMTs play some role in initiating the MRF transcriptional program or in regulating the transition from SC quiescence and proliferation to differentiation. However, the precise mechanisms regulating methylation/demethylation are still elusive.

#### Histone modifications

In QSCs, the transcription factor *PAX7* is expressed due to TrxG activity composed of MLL1/2, ASH2L, WDR5, and RBBP5 subunits [128], while modulators of cell cycle progression and other transcription factors responsible for myogenic differentiation, such as myogenic differentiation 1 (*MYOD*) and myogenin (*MYOG*), remain silenced (Fig. 4a) [129]. However, it was concluded that QSCs are not in a dormant state but rather are primed for activation and thus differentiation in response to external stimuli [130], such as muscle damage observed in DMD patients. At the chromatin level, it was shown that in QSCs permissive H3K4me3 marks can be found not only in actively transcribed but also in inactive genes, to be transcribed at a later time [130]. Particularly, a large number of genes, including *MYF5*, *MYOD,* and serum response factor (*SRF*, a negative transcription regulator of SC differentiation), were found to have opposing H3K4me3 and H3K27me3 marks at the transcription start sites due to the respective TrxG and Ying-Yang 1 (YY1), which recruits the EZH2 subunit (putatively the entire PRC2 complex), activities (Fig. 4a) [130, 131]. It is important to note that not all factors are regulated in the same manner as, e.g., muscle-specific regulatory factor 4 (*Mrf4*) promoter is devoid of active H3K4me3 or repressive H3K27me3 marks [132]. Moreover, somewhat undermining the role of epigenetic regulation, others noted *Myf5* transcripts already in QSCs; however, sequestered inside the cytoplasmic messenger ribonucleoprotein granules as a result of *miR-31* expression and phosphorylation of eIF2 [133, 134]. Following SC activation, the granules are disassembled, leading to Myf5 protein synthesis [133]. Similarly, *MyoD* transcripts were shown to be blocked in QSCs by Staufen 1, a regulator of mRNA localization, stability, and translation [135]. Loss of this repression enables MyoD translation and its accumulation that triggers the myogenic program [135].

*MYOG* expression in QSCs is halted by the PRC2 and YY1 complex and HDACs (Fig. 4a), which interact with chromatin through association with transcription factors and methyl MBD proteins [131, 136–139]. In contrast, *MYF5* repression and *MYOD* repression occur not only through the PRC2 complex and removal of acetylation marks carried by HDACs, but it is also induced by the SUV39H1-driven addition of H3K9me2/3. The expression of *MYOD* is furthermore negatively regulated by a complex array of proteins that contains: an inhibitor of differentiation (ID), the NAD-dependent histone deacetylase activity of Sirtuin 2 (Sir2) [140], a histone acetyltransferase PCAF, as well as the myocyte enhancer factor-2 (MEF2), which also plays a role in recruiting HDACs, and Suv4-20h1/h2 (Fig. 4a) [141, 142]. Importantly, SCs deficient in Suv4-20h1/h2 and its associated H4K20me2/3, have a strong reduction of heterochromatin, which leads to the abnormal regulation of *Myod* expression, premature activation of SCs, and impaired long-term skeletal muscle regeneration [143].

Interestingly, the total levels of PRC2, EZH1, and EZH2 subunits differ during myogenesis [137, 144]. Studies in mice have shown that inactivation of the Ezh2 subunit in SCs results in a lower number of these cells and diminished regenerative potential following muscle-induced injury, attributed to the failure of SCs to proliferate and self-renew [136, 145]. In contrast to the well-defined functions of EZH2, the role of EZH1 is still unclear in QSCs [137].

#### Noncoding RNAs

miRNAs also maintain the specific epigenetic state of chromatin, necessary to keep SCs in quiescence. Castel et al. [145] reported a massive downregulation of miRNA expression during activation of QSCs isolated from mice. Similarly, others indicated that miRNAs have higher expression in QSCs compared to ASCs. This indicates that the quiescent state of SCs is actively suppressed by miRNAs [145]. Specifically, miR-195/497 and miR-489 as well as miR-27b, miR-489, miR-31, and miR-195/197 were identified as key regulators of the SC transition in these two phases [146]. Interestingly, the above observations based on experiments in mice are contradictory to the conclusion of Koning et al. [147], who observed that in human QSCs all miRNAs are downregulated and therefore have minimal regulatory activity. Other miRNAs that were indicated in regulating SC quiescence in adult resting muscles are miR-127 and miR-379. It was shown that their robust expression corresponds to an increase in *Pax7* expression and reduced commitment towards differentiation [145].

A recent increase in interest in the field of lncRNA yielded a few reports that indicate the involvement of these molecules in QSCs. Particularly, high expression of lncRNA H19 was noted, indicative of its involvement in the maintenance of the QSC pool [148]. Furthermore, lncRNA, named Uc.283+ A, might be another key regulator of quiescence, as it can block the formation of miR-195 [149], which, as mentioned above, is needed to maintain the undifferentiated state of SCs.

### Epigenetic control of SC activation and myoblast proliferation

In response to damage, QSCs undergo activation and divide. Symmetric divisions lead to self-renewal of PAX7+/MYF5-/MYOD-/MYOG- QSCs (Fig. 1b) [150]. In contrast, the asymmetric division results in one cell that returns to the quiescent state (PAX7+/MYF5-/MYOD-/MYOG-), while the other (PAX7+/MYF5+/MYOD+/MYOG-; myoblast) proliferates, differentiates, and fuses to form multinuclear myotubes [150]. In this context, myoblasts produce regulatory proteins, including MRFs, such as MYF5, MYOD (required for myoblast specification) (Fig. 4b), as well as MYOG and MRF4 expressed in the early and subsequent stages of differentiation, respectively (Fig. 5a, b). It is worth noting that the proper expression of *MYOD* and *MYOG* depends on the phosphorylation state of MEF2 (Figs. 4 and 5a) [151]. Ultimately, during terminal differentiation, proteins characteristic for mature skeletal muscle such as myosin heavy chain (MYHC), creatine kinase M-type (CKM), and α-actinin 1 (ACTN1) are generated (Fig. 5c) [152–154].

During symmetric and asymmetric divisions of QSCs, the *PAX7* promoter is active, holding transcription permissive chromatin marks by the action of the TrxG complex (Fig. 4a) [155]. The study by von Maltzahn et al. [156] has revealed that PAX7 is a crucial player in the transcriptional regulation of SCs, as in the *Pax7*-deficient mice the population of SCs was completely absent, leading to muscle atrophy and premature death. This phenotype might partially stem from the fact that PAX7 is involved in the repressive regulation of *MYOD* by increasing expression of *Id* in cells undergoing self-renewal following asymmetric division [157]. Interestingly, PAX7 also triggers the synthesis of MYF5 in cells committed to myogenesis. Specifically, upon CARM1-mediated methylation of multiple arginine residues in the N terminus, PAX7 recruits TrxG to regulatory enhancers and the proximal promoter of *MYF5* through direct interaction with MLL1/2 HMT (Fig. 4b) [158–160]. This activation occurs via PAX7 binding to different sites in the *MYF5* promoter in a two-step manner. First, PAX7 binds to a site located in the enhancer marked by H3K4me2 [161], and then a strong H3K4me3 is induced, establishing a transcriptionally active domain. In addition to *PAX7* and *MYF5*, proliferating myoblasts express *MYOD*, which inhibits the cell cycle and induces myoblast differentiation. In this context, *MYOD* expression starts with the detachment of HDACs and the PRC2 complex, and through the involvement of TrxG (H3K4me3), which is recruited by phosphorylated MEF2, as well as SRF, which binds to the serum response element (SRE) [162, 163]. As a consequence, the PCAF-p300/CBP and SWI/SNF chromatin remodeling complexes bind to the *MYOD* promoter and initiate transcription (Fig. 4b). Additionally, noncoding miRNAs are also involved in myoblast proliferation. For instance, miR133a enhances the proliferation of SCs by repressing the SRF (Fig. 7a) [164].

### Epigenetic control of myoblast differentiation

The ability of myoblasts to differentiate into functional multinucleated myofibrils requires coordinated changes in the expression of muscle-specific genes [165]. The MYOD levels are highest at the end of myoblast proliferation (Fig. 4b) and in the initial stage of differentiation (Fig. 5a) as a result of *PAX7* repression via H3K27me3 catalyzed by PRC2 (YY1-EZH2 complex) (Fig. 5a) [157, 166, 167]. Simultaneously, the high content of MYOD contributes to an increase in the production of MYOG (Fig. 5a), which in return inhibits *MYF5* expression by repressive H3K27me3 marks (YY1-EZH2 complex and other unknown factors) (Fig. 5a) [168]. Furthermore, the combined action of MYOD and MYOG leads to the expression of genes characteristic for late differentiation, such as *MRF4* (Fig. 5b), which allows for the formation of muscle fibers [169], and then, the MYOD and MYOG levels decline (Fig. 5b) [170]. In mature myofibers, the level of MRF4 remains high [171], and proteins characteristic for mature skeletal muscle such as MYHC, CKM, and ACNT1 are generated (Fig. 5c) [152–154].

#### Removal of repressive epigenetic marks

During differentiation, HDACs leave the promoters of muscle-specific genes, e.g., *Myog*, to enable the recruitment of transcription factors such as phosphorylated *Mef2* and *Myod*, which in turn recruit the SWI/SNF chromatin remodeling complex and HATs leading to active transcription (Fig. 5a) [172]. Several mechanisms are known that allow HDACs to leave the gene promoters, including the reduction of their expression [172]. More specifically, upon myoblast differentiation, the disruption of the MYOD-HDAC I complex is observed [173] and the nuclear-to-cytoplasmic translocation of HDAC II occurs, thereby releasing the inhibitory constraints of MEF2, which activates the expression of muscle-specific genes [141, 174]. Additionally, upon reception of the differentiation-promoting signals, the NAD+/NADH ratio decreases, and as a consequence, inhibition of HDAC III (SIRT) and an increase in PCAF-p300/CBP complex activity occurs, which induces acetylation of histones in genes, such as *MYOD* (Figs. 4b, 5a) and *MEF2* [175].

Activation of *MYOG* is also dependent on decreases in the PRC2 HKMT activity [176]. The total levels of PRC2 decrease significantly as myogenesis progresses, and correspondingly, the H3K27me3 mark is lifted from the *MYOG* promoter [176]. Additionally, it was also noted that PRC2-EZH1 replaces PRC2-EZH2 on the *MYOG* promoter (Fig. 5a), a process that is necessary to guarantee its activation in post-mitotic myotubes signified by the fact that the depletion of EZH1 negatively affects muscle differentiation and the ability of MYOD to regulate *MYOG* [177]. The Ezh2 to Ezh1 switch has been attributed to the mitogen- and stress-activated protein kinase 1 (MSK1)-mediated phosphorylation of histone H3 at serine 28 (H3S28ph) on, among others, the *Myog* regulatory region promoter [177].

Moreover, the loss of repressive histone marks from *MYOG* is also due to the removal of trimethylation of H3K27 by ubiquitously transcribed X chromosome tetratricopeptide repeat protein (UTX) activity (Fig. 5a), which belongs to the family of JmjC HDMs [178]. UTX acts in complex with several proteins, including mixed-lineage leukemia 4 (MLL4), the HMT subunit of TrxG [179, 180]. In turn, the removal of the repressive H3K9me2/3 marks from the *Myog* promoter [181] is catalyzed by HDM—JHDM2A (KDM4A) that also belongs to the JmjC family (Fig. 5a) [182] as underlined by the study revealing that knockdown of this enzyme leads to the significantly decreased levels of *Myog* expression [181]. Summarizing, the coordinated PRC2-EZH2 and PRC2-EZH1 switches as well as the activity of specific enzymes work together to remove inhibitory marks from the promoters of muscle differentiation-specific genes.

#### Addition of permissive epigenetic marks

*MYOG* activation is initiated upon MEF2 phosphorylation and its association with the ASH2L and MLL2 subunits of TrxG. This complex then binds to the *MYOG* promoter, catalyzing the H3K4me3 mark (Fig. 5a). Tri-methylation of H3K4 is also catalyzed by another HMT, SET7 (Fig. 5a), as underlined by siRNA knock-down experiments [183]. Apart from the acquisition of permissive histone marks, transcription initiation also requires the concerted recruitment of the chromatin remodeling complex SWI/SNF (Fig. 5a). The complex facilitates the binding and formation of the RNA polymerase II preinitiation complex and transcriptional elongation [184, 185] through recognition of acetylated lysines on histone tails by ATPase subunits BRG1 or BRM [186]. Interestingly, MYOD physically associates with the SWI/SNF complex on regulatory elements of MYOD-target genes, including *MYOG*, ensuring their active transcription (Fig. 5a) [187].

The combined action of MYOD and MYOG leads to the expression of genes characteristic for late differentiation, such as *MRF4* (Fig. 5b), which allows for the formation of muscle fibers [169]. In this context, the permissive *MRF4* marks are catalyzed by TrxG (H3K4me3) and by the PCAF-p300/CBP complex (acetylation). In the late differentiation, the levels of MYOD and MYOG decrease in response to the G9a HKMT-mediated H3K9me2 mark [188], the H3K27me3 addition by PRC2 (YY1-EZH2 complex) and probably due to other undiscovered factors (Fig. 5b). Conversely, the level of MRF4 is maintained at a high level [189] and proteins characteristic for mature skeletal muscle such as MYHC, CKM, and ACTN1 are generated (Fig. 5c) [152–154].

#### Noncoding RNA

miRNAs, including muscle-specific miRNAs (myomiRs), are one of the most important players orchestrating the processes of myoblast proliferation and myogenic differentiation [190]. In particular, myomiRs miR-1 and miR-206 target a subunit of DNA polymerase alpha complex, thus promoting differentiation by DNA synthesis inhibition. Other miRNAs involved in myogenesis involve, e.g., miR-29, a negative regulator of YY1 [191], or miR-214 [192] and miR-26a [193] that play crucial roles in the repression of PRC2 by targeting the *Ezh2* mRNA. Moreover, miR-206 and miR-29 negatively regulate *HDAC4* expression [194] and, hence, reduce the total levels of HDACs and in turn MYF5 as well as MYOG [195]. On the contrary, some of the described miRNAs are regulated by myogenic transcription factors that play pivotal functions in myogenesis. Specifically, *MYOD* overexpression translates into an increased concentration of miR-206 and enhanced myoblast differentiation capacity due to the MYOD direct binding to the miR-206 promoter [196]. Also, increasing amounts of MYOG and MYOD were found in regions upstream of miR-133, miR-1, and miR-206 [197], which suggests the involvement of MYOG and MYOD in the regulation of these miRNAs.

LncRNAs have been also shown to regulate myogenic differentiation. For instance, lncRNAs myogenic differentiation 1 (lncMyoD) is activated by MyoD [198]. Upon myoblast differentiation, lncMyoD accumulates and interacts with Igf2 mRNA-binding proteins (IMPs) to inhibit genes promoting myoblast proliferation, such as cyclin G1 (*Ccng1*) or c-Myc. As a result, myoblasts can exit the cell cycle and differentiate [198]. Another example is the long intergenic noncoding RNA activator of myogenesis (lincRAM), whose expression is also regulated by MYOD [198]. However, in this case, lincRAM directly interacts with MYOD and enhances its activity by promoting the assembly of the MYOD-BAF60c-BRG1, SWI/SNF complex which, in turn, remodels the chromatin of MYOD-target genes (e.g. *MYOG*), enabling their subsequent transcription [198]. Furthermore, recent data indicate that lncRNA Myoparr is an essential positive regulator of the myogenic process. It was shown that this *MYOG* promoter-associated lncRNA interacts with the transcriptional coactivator of MyoD, DEAD-Box Helicase 17 (Ddx17), and regulates binding of the latter to PCAF, activating *MYOG* transcription [199]. Additionally, lncRNA Irm is upregulated during myogenesis, promoting myogenic differentiation, while its inhibition has the opposite effect. Interestingly, lncRNA Irm blocks regeneration following cardiotoxin-induced muscle damage in mice and regulates the expression of myogenic genes through direct binding to MEF2, which in turn mediates MYOD/MEF2 interaction with target genes [200]. Another lncRNA implicated in myogenic differentiation involves a long intergenic non-protein coding RNA, muscle differentiation 1 (lncRNA lincMD1), which is localized in the cytoplasm and acts as a natural decoy for miR-133 and miR-135 [192]. Likewise, metastasis-associated lung adenocarcinoma transcript 1 (*Malat1*) lncRNA also acts as a sponge for miR-133. Particularly, its presence has been associated with the inhibition of SRF expression that allows for myoblast terminal differentiation [164].

### Cell signaling control of epigenetic changes during muscle differentiation

**Fig. 6:**
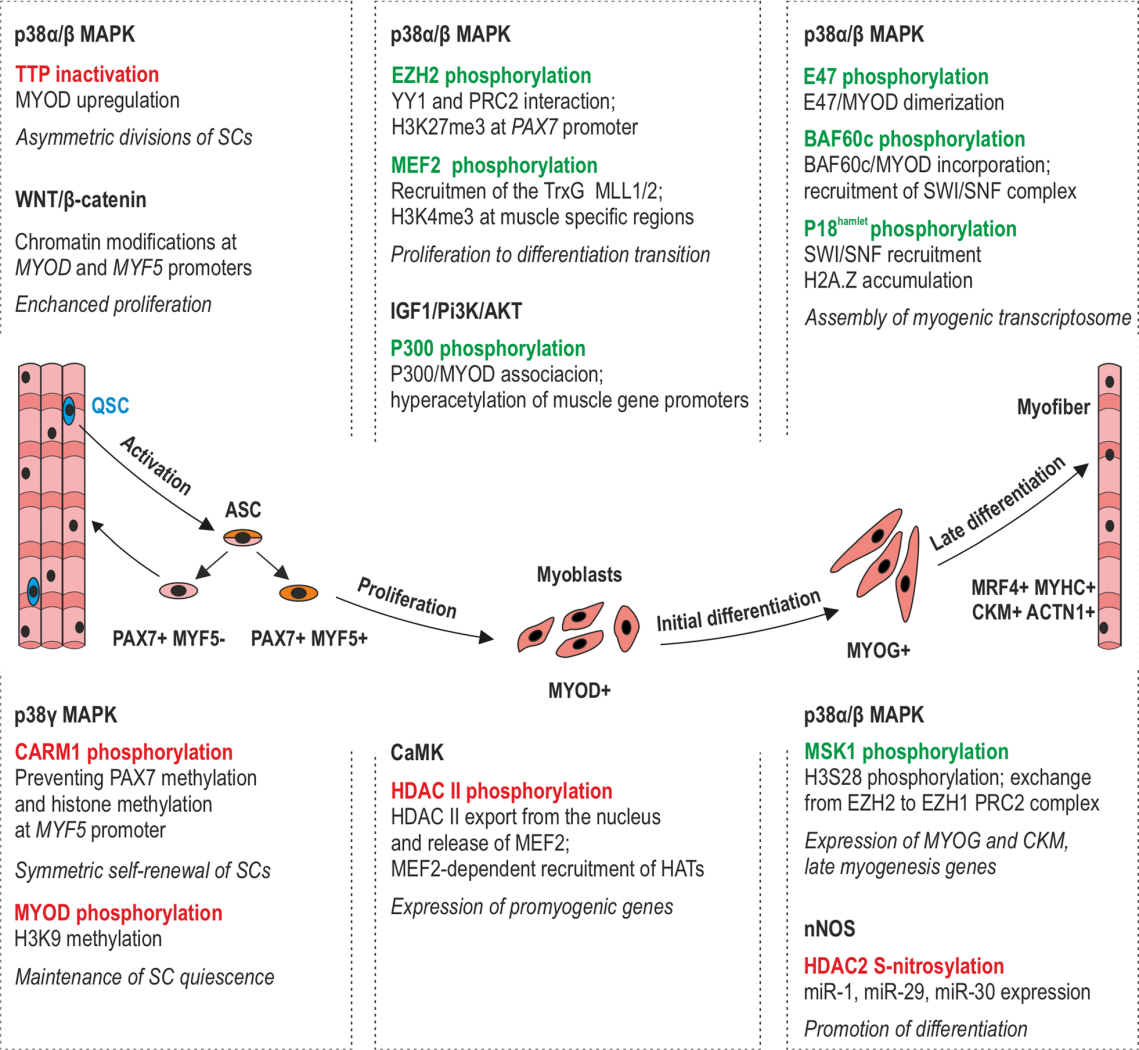
Cell signaling pathways in the epigenetic regulation of myogenesis. p38α/β MAPK, p38γ MAPK, IGF1/Pi3K/AKT, CaMK, Wnt/β-catenin, and nNOS signaling either inactivates (marked red) or activates (marked green) downstream targets, dependent on the stage of the muscle formation. The consequences following particular signaling events and the final impact on myogenesis (in italics) are listed below the name of each signaling pathway

The dynamic changes in the genome expression landscape of SCs during their activation and division as well as myoblast proliferation and differentiation are coordinated by extracellular signals that manage multicellular processes in response to microenvironmental requirements. These signals not only regulate genome expression through modulating the levels of transcription factors but also by influencing DNA structural alterations, e.g., by recruiting chromatin modifier enzymes (Fig. 6) [28, 201]. The following signaling pathways have been identified as epigenome regulators in the context of muscle differentiation: p38 MAPK, IGF1/Pi3K/AKT, Wnt,  Ca^2+^/calmodulin-dependent protein kinase (CaMK), TNFα, and nuclear factor kappa-light-chain-enhancer of activated B cells (NFκB). Nevertheless, it is important to note that despite the growing number of studies, our knowledge is still limited and requires further exploration. Furthermore, while cell signaling influences epigenetic modifications, it can itself be tightly controlled by epigenetic events. As an example, the Notch pathway is implicated in the quiescence of SCs, the proliferation of myoblasts, and the transient inhibition of terminal differentiation of myoblasts into mature myofibers. Such a wide range of controlled processes the Notch pathway owes to a variety of Notch receptors and ligands required to activate downstream signaling [202]. Expression of Notch receptors and ligands has to be precisely regulated in a time- and space-restricted manner. Gene expression profiling and epigenetics studies performed by the Terragni et al. [202] revealed significant hypomethylation and very high enrichment of 5-hydroxymethylcytosine in myoblasts, myotubes, and skeletal muscle at intragenic or intergenic regions of some Notch receptors and ligand genes. Their results suggest that hypomethylation and/or hydroxymethylation of the Notch pathway genes is the mechanism of epigenetic regulation of Notch signaling activity.

Exercise or muscle injury activates SCs via Wnt signaling. Wnt/β-catenin pathway modifies chromatin at the promoter regions of *Myod* and *Myf5,* activating their expression and, as a consequence, enhancing SC proliferation. In fact, Wnt signaling, which is further involved in the regulation of different myogenesis stages (expansion, proliferation-to-differentiation transition, cell motility), is under the epigenetic control of p38α MAPK [203, 204], the most studied signaling pathway in the context of muscle formation. In mammals, the family of p38 MAPK consists of four kinases, p38α, p38β, p38γ, and p38δ, all phosphorylated and activated by MAPK kinases (MKK). p38α is the most abundant isoform that was found to take part in the epigenetic regulation at various stages of muscle development and via different downstream mechanisms [204]. Upon SC activation, p38α/β MAPK signaling promotes asymmetric divisions and myogenic commitment via promoting the accumulation of MyoD. This occurs in the following manner: p38α/β MAPK phosphorylates MAPKAP2, which in turn inactivates, via phosphorylation, tristetraprolin (TTP). TTP is the mRNA decay factor, which in the active state promotes MyoD mRNA decay, leading to the maintenance of SCs quiescence [205].

The quiescence of SCs and their self-renewal via symmetric divisions is also controlled by p38γ MAPK, which phosphorylates Carm1, a process that prevents its nuclear localization and methylation of Pax7. Otherwise, methylated Pax7 recruits TrxG MLL1/2 HMT complex to regulatory enhancers and promoter regions of *Myf5*, leading to an “open mark” H3K4 methylation and *Myf5* expression that favors asymmetric cell division (Fig. 4b) [159]. In the later stage of myogenesis, Carm1 additionally recruits SWI/SNF complex to promoters of muscle-specific genes like desmin (*DES*) or *CKM* [206]. p38γ MAPK signaling was found to contribute to the maintenance of SCs quiescence also via phosphorylation of MyoD and establishing MyoD and Suv39h1 HKMT on *MYOG* promoter, followed by methylation of H3K9, which induces transcriptionally repressive chromatin and prevents premature myoblast differentiation (Fig. 4b) [207]. Furthermore, p38γ MAPK activity was noticed at the proliferation and differentiation stages of myogenesis. However, its exact mechanistic involvement in the temporal patterning of gene expression remains unknown in these processes [207].

p38α MAPK phosphorylates EZH2, which plays a crucial role at the stage of transition from myoblast proliferation to its differentiation. TNF-activated p38α MAPK promotes PRC2 and YY1 interaction via phosphorylation of PRC2 EZH2 enzymatic subunit, which effects in repressive chromatin in the *Pax7* promoter region (Fig. 5a) [208]. At the onset of differentiation, p38 MAPK phosphorylates P18hamlet, a subunit of SNF2-related CBP activator protein (SRCAP). As a consequence, SWI/SNF transcription-activating complex localizes to the *MYOG* promoter, which is accompanied by H2A.Z histone accumulation and expression of muscle-specific genes [209]. However, the mechanisms of epigenetic control via the p38α MAPK pathway are much more complex. p38α MAPK influences the epigenetics of muscle cells via interaction with various transcription factors, especially MyoD and the binding partner for MRFs (E47) [210]. Phosphorylation of E47 initiates the dimerization of E47 with MyoD and further localization of this heterodimer at myogenic loci [211]. Additionally, MyoD binding to target genes is facilitated by p38α MAPK-dependent phosphorylation of Baf60c, which results in MyoD-Baf60c incorporation into SWI/SNF chromatin remodeling complex and expression of MyoD-controlled genes [187]. In differentiating myoblasts, p38α/β activates Msk1, which in turn phosphorylates histone H4S28, implicated in Ezh2-containing PRC2 complex displacement of *MYOG* and *CKM* genes. Meanwhile, PRC2-Ezh2 is replaced by the PRC2-Ezh1 complex, precisely activating their expression [177, 203]. The p38α signaling pathway also regulates the EZH2 levels at the early stages of muscle differentiation, leading to its degradation through the proteasome, more specifically, by the E3 ubiquitin ligase Praja1 (*PJA1*) [212]. Transcription of *MYOG* or *CKM* in proliferating myoblasts is also activated via recruitment of Ash2L/MLL2-containing TrxG HMT complex by phosphorylated Mef2, a downstream target of the p38α/β MAPK pathway [213, 214]. This complex catalyzes tri-methylation of H3K4, a permissive epigenetic mark. Such a variety of actions allows for the speculation that p38α/β plays a bidirectional role in the proliferation-to-differentiation transition, both silencing genes responsible for proliferation and promoting the expression of prodifferentiation genes [204].

p38 MAPK signaling control of epigenetics is convergently accompanied by IGF1/Pi3K/AKT pathway. IGF1-activated AKT1 and AKT2 phosphorylates the acetyltransferase p300, which promotes its connection with MyoD and PCAF acetyltransferase. This results in hyperacetylation of muscle gene promoters and chromatin remodeling by the p38 MAPK-recruited SWI/SNF complex [215]. This is consistent with studies showing that a combination of both p300 and PCAF acetyltransferases acts as a strong activator of transcription, unlike PCAF alone that without the presence of p300 is just a moderate inducer [216]. Recruitment of HATs to muscle-specific gene promoters is also induced by CaMK signaling. At the onset of differentiation, CaMK directly phosphorylates members of the class II HDACs, HDAC4 and HDAC5, and forces them to move from the nucleus and release MEF2 from repressing interactions. In turn, MEF2 is potent to associate with HATs and promote the expression of muscle-specific genes [215, 217]. Another histone deacetylase, HDAC2 that belongs to the class I HDACs, loses affinity to chromatin and is released from specific miRNA promoters that enables their expression after NO-induced S-nitrosylation [218, 219]. Moreover, miR-133 enhances proliferation (via interaction with Akt pathway) [220], while miR-29 (downregulating Akt signaling) together with miR-1 and miR-30 promotes differentiation of myoblasts [220–223].

## Epigenetic regulation of gene expression in Duchenne muscular dystrophy

Besides the well-established function as a mechanical anchor between the cytoskeleton and the ECM of myofibers, the DGC is now considered as a scaffold for signaling molecules in various cell types, including muscle fibers and SCs. Particularly, the absence of dystrophin in SCs of DMD patients has been associated with the signal transmission loss between the plasma membrane and the nucleus, leading to the SC aberrant epigenetic transcriptional activation and impaired regenerative ability [224–226].

### Dystrophin deficiency in muscle regeneration

Dystrophin deficiency in ASCs, primed to divide asymmetrically, reduces the levels of the DGC and induces aberrant polarization of structural and signaling proteins. This leads to impaired signal transduction and transcriptional activity in the newly generated cells [227]. The defects in polarization, centrosome amplification, and prolonged cell divisions of dystrophic ASCs have been attributed to the loss of dystrophin interaction with MARK2 [228–231] as well as downregulation and mislocalization of another DGC component, β-syntrophin. Generally, β-syntrophin interacts with p38γ, modulating CARM1-mediated activation of *MYF5* in the opposite cell that undergoes myogenic differentiation [232], while dystrophin deficiency leads to impaired polarization of p38γ, enhanced phosphorylation of CARM1, and reduced ability of *MYF5* to be activated by PAX7 [233]. Furthermore, in DMD, elevated levels of TNFα and NFκB were found to diminish the regenerative potential of SCs and this is connected to epigenetic silencing of *Notch-1* via hypermethylation of its promoter region [234]. What is more, the lack of dystrophin causes loss of nNOS binding sites and, as a consequence, reduces nNOS sarcolemmal localization [235]. This results in diminished NO signaling and, in turn, a decrease in NO-dependent S-nitrosylation of HDAC2. Importantly, restoration of NO-signaling-dependent inhibition of HDAC2 shows beneficial effects in dystrophic mice [236].

A recent study uncovered a crosstalk between fibro-adipogenic progenitors (FAPs) and the myogenic lineage, which sheds more light on adipocyte and myofibroblast accumulation in dystrophic skeletal muscle. The data indicate that soluble molecules released by myogenic progenitors activate the PI3K/Akt pathway in FAPs, stimulating their proliferation, while myotubes induce their differentiation through the secretion of pro-fibrogenic and anti-adipogenic factors [237]. As in DMD patients proliferation and differentiation of myogenic cells are disrupted, this results in excessive FAP proliferation [237] and their transformation into fibroadipocytes. Altogether, these processes mediate fat deposition and fibrosis in skeletal muscle [238]. A few signaling pathways associated with these pathological alterations have been described. Namely, Notch-mediated modulation of FAP adipogenesis was found compromised in FAPs from the *mdx* mouse, an animal model of DMD, supporting a model whereby the synergistic cooperation of Notch with other anti-adipogenic signals plays essential roles in the regulation of FAP adipogenesis in both healthy and dystrophic muscle [239]. Additionally, a recent report by Mázala et al. [240] indicated that muscle damage in *mdx* mice contributes to an increase in TGF-β activity accompanied by increased accumulation of FAPs, which leads to muscle fibrosis. Nevertheless, it is important to mention that although inhibition of TGF-β signaling blocked the accumulation of FAPs, it did not induce muscle regeneration [240, 241]. Communication between myogenic cells and FAPs expresses through appropriate epigenetic modifications, and FAPs are an important intervention target aimed at restoring the balance between skeletal muscle regeneration and degeneration in DMD. Specifically, histone deacetylase inhibitors (HDACi) are used to promote muscle gene expression and skeletal myogenesis. In this context, HDACi appears to selectively upregulate genes that are enriched in permissive H3K4me3 marks or marked by bivalent domains (H3K4me3/H3K27me3) [242].

At the epigenetic level, HDACi induce upregulation of MYOD and BAF60C (a subunit of SWI/SNF complex) and lead to the upregulation of miR-1, miR-133, and miR-206. These in turn target the alternative BAF60 variants, BAF60A and BAF60B, ultimately leading to promyogenic differentiation and simultaneous suppression of the fibro-adipogenic phenotype [243]. Interestingly, Saccone et al. [243] demonstrated that HDACi delivery by intraperitoneal injection into young *mdx* mice promotes myogenic differentiation of FAPs, while such an effect was not observed in wild-type or old *mdx* mice. This indicates that FAPs may support the activity of SCs or promote fibro-adipogenic degeneration and that the latent FAP myogenic phenotype may be induced in response to regenerative signals.

**Fig. 7:**
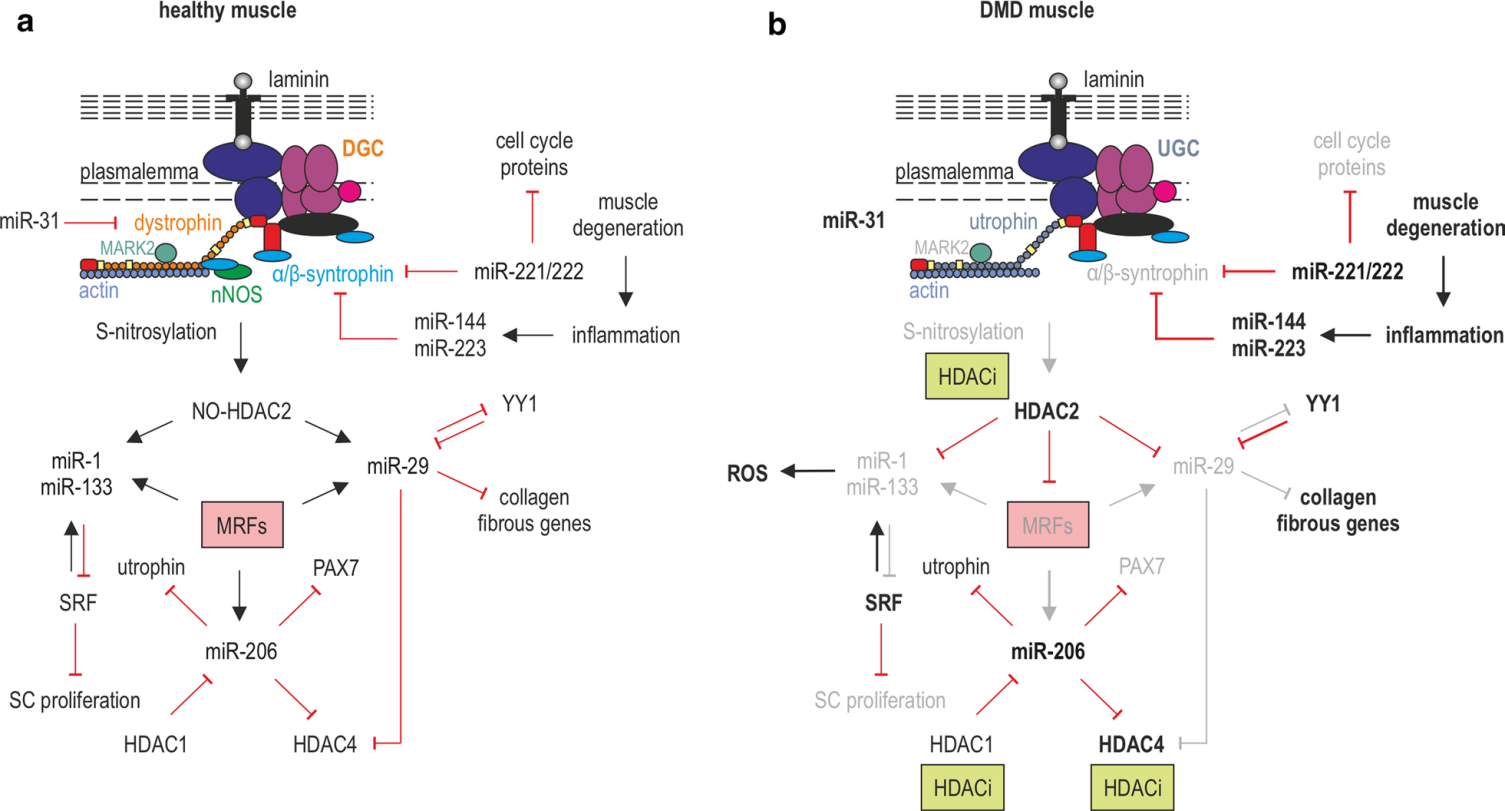
Dystrophin-nNOS signaling in epigenetic control of muscle differentiation. **a** The dystrophin-nNOS signaling regulates the epigenetic profile of myogenesis via S-nitrosylation of HDAC2 (class I HDAC), which affects gene expression through changes in histone acetylation. miR-221/222 are involved in the inhibition of cell cycle proteins and miR-222 targets β-syntrophin, while miR-31 temporarily targets dystrophin. Transcription of miR-1 and miR-133 is controlled by the HDAC2 S-nitrosylation state, regulated by nNOS activity. Also, miR-133 targets SRF during proliferation, which in a self-regulating manner promotes miR-133 expression, and miR-29 is coregulated by the HDAC2 S-nitrosylation state and YY1, while miR-206 is regulated by MRFs and the HDAC1 activity, and supports cell cycle inhibition by targeting PAX7. **b** In DMD, the epigenetic differentiation network is disturbed due to the absence of dystrophin-α/β-syntrophin-nNOS signaling and interrupted HDAC2 S-nitrosylation. As a consequence, a decrease in the levels of miR-133/1 and miR-29, followed by inhibition of muscle differentiating genes and ROS generation is observed. Also, diminished amounts of miR-29 levels correlate with an increase in collagen and fibrotic tissue. The miR-206 level is higher, resulting in an imbalance between proliferative and differentiated states. Additionally, miR-144 and miR-223 are also observed following an increase in inflammation and muscle degeneration. miRNA and protein names marked in bold and grey indicate their upregulation and downregulation, respectively; black arrows mark activation while red and grey blunt lines inhibition and reduction in inhibition, respectively. HDACi targeting HDACs and the corresponding processes are marked in yellow boxes

Concomitantly, fibrosis can be stimulated not only by FAPs but also by miRNAs, e.g., miR-21 and miR-29 that play opposing roles in DMD muscle fibrosis [244]. MiR-21 is involved in the pro-fibrotic effects induced by TGF-β treatment [245], and conversely, miR-29 downregulates the expression of ECM components such as collagen and elastin [246]. In particular, miR-29 is downregulated in *mdx* mice, a process that has been linked to fibrosis and impaired muscle regeneration [246]. At the epigenetic level, miR-29 promotes myogenesis by direct inhibition of a negative regulator of muscle genes, YY1 [191], as also shown by Zanotti et al. [244] (Fig. 7). The other miRNA involved in the pathogenesis of DMD is miR-206, which targets utrophin mRNA (a paralog of dystrophin) and whose appropriate concentration in skeletal muscle could inhibit the development of DMD [247, 248].

## Epigenetic therapies in Duchenne muscular dystrophy

Myogenesis is coordinated by a complex set of epigenetic mechanisms that include DNA methylation, histone modifications, and rncRNA expression, and as such, targeting epigenetic modifiers is a promising pharmacological approach, opening new therapeutic avenues in muscle diseases. Particularly in DMD, one could envision changing the epigenetic status of SCs or myocytes to increase their regenerative potential.

Altered cell signaling poses a wide range of potential therapeutic strategies for DMD. Some pharmacological therapies are based on modulation of cell signaling, e.g., NO administration, stimulation of IGF-1, inhibition of TGFβ, or modulation of NFκB and TNFα pathways. However, changes in signaling pathways entail a risk of undesired side effects as distinct signaling pathways are differently activated depending on the muscle type [249] and differentiation stage. For this reason, epigenetic drugs aiming to modulate targets of signaling pathways seem to be a safer therapeutic approach [250]. Pharmacological inhibition of HDACs by HDACi enhances histone acetylation in the promoters of muscle-specific genes leading to their increased expression (Fig. 7b) [251]. However, it should be noted that HDACi acts systemically, affecting acetylation, and thus increases the expression of many genes. Indeed, HDACi has been associated with side effects such as nausea, neutropenia, thrombocytopenia, or ventricular arrhythmia [252].

Iezzi et al. [253] reported enhanced histone acetylation in the regulatory elements of *Myod* in wild-type myoblasts following HDACi treatment [253]. Moreover, HDACi delivery to myoblasts before the onset of differentiation upregulated *Myf6* and *Myog* [254]. Interestingly, HDACi also increased follistatin expression [255], which downregulates myostatin, a major inhibitor of skeletal muscle regeneration [256, 257], and increased myoblast fusion [258]. In all, the results obtained in wild-type myoblasts highlight the potential of HDACi as a treatment having a positive effect on muscle regeneration in DMD.

HDACi ITF2357 (Givinostat) is the first epigenetic drug tested in preclinical studies in *mdx* mice [255, 259] and clinical trials [260]. Preclinical studies revealed that after 3.5 months of Givinostat treatment, *mdx* mice exhibited increased myofiber mass and size as well as restored muscle force to the levels observed in wild-type mice. At the same time, a decrease in the cellular inflammatory infiltrate, reduction in the formation of fibrosis, and accumulation of fat tissue were observed [259]. In another preclinical study, Givinostat alleviated the morphological and functional phenotypic consequences of dystrophin deficiency in *mdx* mice [255]. The success of these studies paved the way for I/II clinical trials with children affected by DMD (ClinicalTrials.gov Identifier: NCT01761292) [260]. Phase I and phase II clinical trials were conducted on 20 boys aged 7 to < 11 years. The current results indicate that long-term (over 1 year) treatment of Givinostat results in an increased fraction of muscle tissue as well as a decreased amount of fibrotic tissue and also reduced necrosis and fatty replacement [260] compared to untreated boys aged 7 to 10 years [261, 262]. Summarizing, Givnostat is the first pharmacological treatment shown to produce beneficial histological effects in muscle samples from DMD patients [260]. Based on these promising results, the clinical trial has been extended to the III phase (ClinicalTrials.gov Identifier: NCT03373968). Besides, one more clinical trial is currently underway to evaluate the efficacy and safety of Givinostat in ambulant DMD patients (6 to 17 years) (ClinicalTrials.gov Identifier: NCT02851797).

Trichostatin A (TSA) is another promising HDACi used to enhance myogenic regeneration. For example, in wild-type myoblasts (murine and human origin), TSA increased their fusion and favored myogenic differentiation but without leading to hypertrophy (fibrosis caused by an increased amount of fat components) of preformed myotubes [253, 254]. Besides, intraperitoneal TSA delivery to *mdx* mice increased utrophin expression and improved the structure and function of skeletal muscles [263]. TSA also ameliorated pathological alterations in a zebrafish model [264], which is an outstanding model for screening and evaluating novel drug therapies, e.g., in DMD [265]. In a recently published study, the authors performed a pilot screen of the commercially available Cayman Chemical Epigenetics Screening Library to identify epigenetic molecules that could improve muscle phenotype in the DMD zebrafish model. Interestingly, they proved that a novel combination of HDACi drugs, oxamflatin, and salermide significantly rescued muscle degeneration [266]. In particular, oxamflatin is an HDACi that inhibits HDACs classes I and II and is chemically similar to TSA. On the contrary, salermide belongs to a class III HDACi, which inhibits the NAD+-dependent deacetylases SIRT1 and SIRT2 [267] and represents a new class of HDACi in DMD treatment.

## Conclusion

DMD is a quickly progressing and devastating genetic disorder. A medication that would alleviate the primary symptoms of the disease, i.e., the proper functioning of skeletal muscles and their regeneration, is of utmost need. In this review, we discussed the interplay of various factors that define the specific state of epigenetic homeostasis and contribute to the progression of DMD in skeletal muscles. The described studies show that myogenesis is strictly controlled by interdependent epigenetic pathways; however, it is also characterized by high cellular plasticity, amenable for therapeutic approaches altering the epigenetic status of chromatin. Particularly, HDACi delivery has proven to be an exceptionally effective strategy for restoring the regenerative ability of dystrophic muscles. Although further research is needed in this field, the outcome of the most recent therapeutic advances gives patients hope for a treatment that would significantly alleviate their condition.

## Data Availability

Not applicable.

## Abbreviations

ACTN1: α-Actinin 1
ASCs: Activated satellite cells
BAFs: BRG1-associated factors
*BRG1*: Brahma-related gene 1
*BRM*: Brahma
CARM1: Coactivator-associated arginine methyltransferase
CBP: CREB binding protein
Ccng1: Cyclin G1
circ-BNC2: Circular basonucling 2
circ-QKI: Circular QKI
circRNAs: Circular RNAs
CKM: Creatine kinase M-type
CpG: Cytosine-guanine adjacent sites
CTX: Cardiotoxin
Ddx17: DEAD-Box Helicase 17
DGC: Dystrophin–glycoprotein complex
DMD: Duchenne muscular dystrophy
*DMD*: Dystrophin
DNMT3L: DNMT3-like
DNMTs: DNA methyltransferases
ECM: Extracellular matrix
FAPs: Fibro-adipogenic progenitors
HATs: Histone acetyltransferases
HDACi: Histone deacetylase inhibitor
HDACs: Histone deacetylases
HMTs: Histone methyltransferase
IL-10: Interleukin 10
IL-1β: Interleukin 1 beta
IMPs: Igf2 mRNA-binding proteins
JmjC: Jumonji C domain
lincRAM: LincRNA activator of myogenesis
lncMyoD: LncRNAs myogenic differentiation 1
lncRNA lincMD1: Long intergenic non-protein coding RNA, muscle differentiation 1
lncRNAs: Long noncoding RNAs
LSD1: Lysine-specific histone demethylase 1
Malat1: Metastasis-associated lung adenocarcinoma transcript 1
MARK2: Serine–threonine protein kinase 2
MBD: Methyl CpG binding domain
MEF2: Myocyte enhancer factor 2
miRNA: MicroRNA
MLL1/2: Mixed-lineage leukemia 1/2
MLL4: Mixed-lineage leukemia 4
MRF4: Muscle-specific regulatory factor 4
MRFs: Myogenic regulatory factors
MSK1: Mitogen- and stress-activated protein kinase 1
*MYF5*: Myogenic factor 5
*MYF6*: Myogenic factor 6
MYHC: Myosin heavy chain
*MYOD*: Myogenic differentiation 1
*MYOG*: Myogenin
myomiRs: MiRNAs that are specifically expressed in muscle tissue
ncRNAs: Noncoding RNAs
nNOS: Nitric oxide synthase
NO: Nitric oxide
*PAX7*: Paired box 7
PCAF: P300/CBP-associated factor
PHD: Plant homeodomain
PI3Kinase: Phosphoinositide 3-kinase
piRNAs: PiwiRNAs
*PJA1*: Praja1
PRC2: Polycomb repressive complex 2
PRMTs: Protein arginine methyltransferases
QSCs: Quiescent satellite cells
RITS: RNA-induced transcriptional silencing
rncRNAs: Regulatory noncoding RNAs
ROS: Reactive oxygen species
SAM: S-adenosyl-L-methionine
SCs: Satellite cells
SET: Su(var)3–9, enhancer of zeste, and trithorax catalytic domain
SIRT2: Sirtuin 2
siRNAs: Short interfering
sncRNAs: SMALL noncoding RNA
SRF: Serum response factor
SUV39H1: Suppressor variegation 3–9 homolog 1
SUV4-20: Suppressor of variegation 4–20
SWI/SNF: SWItch/sucrose nonfermentable
TNF-α: Tumor necrosis factor alfa
TRDMT1: TRNA aspartic acid methyltransferase 1
TrxG: Trithorax group
TSS: Transcriptional start sites
UHRF1: UBIQUITIN-like with PHD and ring finger domains 1
YY1: Yin Yang 1
